# Regulatory and Scientific Advancements in Gene Therapy: State-of-the-Art of Clinical Applications and of the Supporting European Regulatory Framework

**DOI:** 10.3389/fmed.2017.00182

**Published:** 2017-10-26

**Authors:** Marta Carvalho, Bruno Sepodes, Ana Paula Martins

**Affiliations:** ^1^Faculdade de Farmácia, Research Institute for Medicines and Pharmaceutical Sciences (iMed.ULisboa), Universidade de Lisboa, Lisboa, Portugal

**Keywords:** advanced therapy medicinal products, gene therapy, Committee for Advanced Therapies, gene delivery vector, drug development

## Abstract

Advanced therapy medicinal products (ATMPs) have a massive potential to address existing unmet medical needs. Specifically, gene therapy medicinal products (GTMPs) may potentially provide cure for several genetic diseases. In Europe, the ATMP regulation was fully implemented in 2009 and, at this point, the Committee for Advanced Therapies was created as a dedicated group of specialists to evaluate medicinal products requiring specific expertise in this area. To date, there are three authorized GTMPs, and the first one was approved in 2012. Broad research has been conducted in this field over the last few decades and different clinical applications are being investigated worldwide, using different strategies that range from direct gene replacement or addition to more complex pathways such as specific gene editing or RNA targeting. Important safety risks, limited efficacy, manufacturing hurdles, or ethical conflicts may represent challenges in the success of a candidate GTMP. During the development process, it is fundamental to take such aspects into account and establish overcoming strategies. This article reviews the current European legal framework of ATMPs, provides an overview of the clinical applications for approved and investigational GTMPs, and discusses critical challenges in the development of GTMPs.

## Introduction

Advanced therapy medicinal products (ATMPs) represent a major class of innovative therapies that differ substantially from traditional therapeutic agents. ATMPs include gene therapy medicinal products (GTMPs), somatic cell therapy medicinal products (sCTMPs) and tissue-engineered products (TEPs). Extensive research is being conducted to study ATMPs as they have the potential to address highly unmet medical needs. In a recent study by Hanna et al., between 1999 and 2015, there were almost one thousand clinical trials investigating ATMPs, mainly in cancer and cardiovascular diseases. More than half of these trials studied sCTMPs, while the other half was equally split between GTMPs and TEPs (1).

Therapeutic products based on the use of genes to prevent or treat diseases are not a new concept and were hypothesized as medicinal products since the discovery of recombinant DNA technology. A high number of diseases have underlying genetic causes, ranging from defects in a single gene (e.g., hemophilia) to more complex disorders affecting multiple genes (e.g., cancer). Replacing the defective gene with a functional copy is the principle of gene therapy (2). Conversely, in China, in 2003, the first worldwide regulatory approval of a GTMP (Gendicine) was a revolutionary milestone as it transformed the previously theoretical concept into a reality (3).

Advanced therapy medicinal products have the potential to be preventive but also curative therapeutic approaches, with an anticipated high price (1). A significant impact in the health system is expected and for this reason broad understanding of ATMPs is fundamental to manage their availability appropriately.

This article aims to (i) provide a regulatory overview of the legal framework in Europe toward granting ATMP marketing authorization; (ii) describe strategic clinical applications, particularly in GTMPs, focusing on currently EU approved medicines as well as in a number of promising investigational treatments; and (iii) explore pre-identified challenges in gene therapy development and post-authorization use.

## European Regulatory Background

### From Directive 2003/63/EC to the ATMP Regulation

Legally, the ATMP concept was first introduced in 2003 through Directive 2003/63/EC where ATMPs were defined as products “*based on manufacturing processes focused on various gene transfer produced bio-molecules, and/or biologically advanced therapeutic modified cells as active substances or part of active substances*” (4). Therefore, TEPs were excluded as medicinal products, leading to ambiguity across Europe. To address this gap, in 2007, Regulation (EC) No. 1394/2007, also known as ATMP Regulation, was created.

The ATMP regulation is considered a *lex specialis* which intends to present a clear definition of ATMPs, outline the marketing authorization requirements and procedures and describe the post-authorization obligations, specifically focusing on efficacy, safety, and risk management.

Advanced therapy medicinal products include GTMPs, sCTMP, TEPs, and combined ATMPs (5). Both sCTMP and TEP are often referred to as cell-based medicinal products (6).

The definition of GTMP can be found in Directive 2009/120/EC amending Directive 2001/83/EC, part IV of Annex I. GTMP are defined as biological medicinal products which include “*an active substance containing or consisting of a recombinant nucleic acid used in or administered to human beings with a view to regulating, repairing, replacing, adding or deleting a genetic sequence. Its therapeutic, prophylactic or diagnostic effect relates directly to the recombinant nucleic acid sequence it contains, or to the product of genetic expression of this sequence*.” GTMP do not include vaccines against infectious diseases (7).

Generally, gene therapy can be divided into two categories: germ line gene therapy and somatic gene therapy. In somatic gene therapy, the genetic material is inserted within the target cells, though the change is not passed on to the next generation, whereas in germ line gene therapy the therapeutic or modified gene will be passed along to the next generation. This is a significant difference, since the current legislation only allows gene therapy on somatic cells (3).

Detailed definition for sCTMP, TEP’s and combined AMTPs can be found elsewhere (5, 7).

### The CAT: A Key Player in Marketing Authorization Application (MAA) for ATMPs

Advanced therapy medicinal products MAA should follow the centralized procedure on a compulsory basis. The benefits of centralized review include overcoming the scarcity of expertise in this area, ensuring a high level of scientific evaluation by a specialized Committee. Since the outcome of the MAA process is applicable to all Member States, the centralized procedure aims at improving market access for these innovative therapies.

Comparing to other medicinal products, the scientific assessment of ATMPs is slightly different as the primary review is performed by the CAT. This is an independent specialist committee which the main responsibility is to review MAA for ATMPs and issue a draft opinion for the CHMP to make a recommendation to the European Commission, which has the final authority to grant marketing authorization (6, 8).

The CAT is lead by an elected chair and includes members of the CHMP, representatives of each EU Member State, patients’ organizations representatives and clinician representatives nominated by the European Commission (6).

Besides reviewing applications for marketing authorization, another of the CAT’s major tasks is to encourage the development of new ATMPs. Several regulatory strategies are currently in place to support ATMP development where the CAT plays a central role, such as the Innovative Task Force, the ATMP Classification, the ATMP Certification, the Scientific Advice and the PRIority MEdicines scheme. Finally, the CAT should also scientifically assist in the elaboration of any documents related to the fulfillment of the objectives of the ATMP Regulation (5, 9).

Obtaining Marketing Authorization Approval of a gene and/or cell therapy product is a worldwide diverse process. Different steps and requirements may be needed depending on the evaluating regulatory body (10). For instance, in the US, gene and cell therapy are considered biologic therapies. Within the Food and Drug Administration (FDA), these products’ primary oversight falls under the Office of Cellular, Tissue and Gene Therapy (OCTGT) which is a division of the Center for Biologics Evaluation and Research.

Initially, an investigational new drug (IND) Application is needed for the investigational use of a biologic. It intends to support clinical use of the investigational product based on quality and non-clinical data. To market a biologic drug product, FDA requires Sponsors to hold an approved biologics license application (BLA). Timelines for evaluation range from 10 to 12 months from filing, depending on the pathway under which the BLA is reviewed. Like the EMA, the FDA has a number of initiatives in place to support the development of Gene and Cell therapies. These include (i) Fast Track designation, (ii) Breakthrough Therapy designation, (iii) Accelerated Approval, and (iv) Priority Review designation. As an example, in case the BLA is evaluated under Priority Review, a reduction to 6-month review time may be granted. Several web-based trainings hosted by OCTGT staff focusing on many regulatory topics can be easily found elsewhere (11) and additional supportive information is available in FDA’s website (12).

## Gene Therapy Medicinal Products

Human gene therapy is based on the simple principle that if a disease is caused by a defective gene, then curing the disease would be as simple as replacing the faulty genetic sequence with a functional copy. Gene therapy consists of using recombinant nucleic acids as the active pharmaceutical ingredient, where the effect is directly related to either the recombinant nucleic acid sequence it contains, or to the product of genetic expression of this sequence (13–16).

### First Steps in Gene Therapy

The first direct human gene therapy trial took place in 1974. In this study, the wild-type Shope papilloma virus was administered intravenously to two female patients suffering from hyperargininemia, an urea cycle disorder, with the intention of introducing the gene for arginase. It was believed that the Shope papilloma virus encoded the gene for arginase activity and that the gene could be transferred by administering the virus to the patients. Unfortunately, the trial was unsuccessful. There was neither a change in the arginine levels, nor in the clinical course of the hyperargininemias (17, 18).

Blaese was the first investigator to conduct a trial using a therapeutic gene (19). In 1990 the FDA approved, for the first time, a gene therapy trial with therapeutic attempt in humans. Two adenosine deaminase deficiency (ADA-SCID) pediatric patients were administered with autologous *ex vivo* modified white blood cells. ADA-SCID is a monogenetic disease leading to severe immunodeficiency where lymphocyte counts are virtually absent. The clinical manifestations of this disease go beyond the immune system and may include deafness, behavioral problems, costochondral abnormalities and hepatotoxicity (20, 21). The cells were modified to express the normal adenosine deaminase gene. Although the treatment was shown to be safe, its efficacy was not fully demonstrated as the patients still required maintenance treatment with enzyme replacement therapy using polyethylene glycol adenine deaminase, and the ADA transduced stem cells were unable to reconstitute the recipient’s immune system. Later on, an ADA-SCID trial was also conducted in Europe (22) and further gene transfer trials were started for several diseases.

No major safety concerns were raised until the unfortunate death of a patient in a gene therapy trial, in 1999, for partial deficiency of ornithine transcarbamylase (OTC). This event took place in the University of Pennsylvania, in Philadelphia. The patient was administered with a very high dose of adenovirus (AdV) carrying the missing gene, his immune system responded immediately and after just a few days the patient died as a result of multiorgan failure (23, 24).

The first country to approve a gene therapy based product for clinical use was China, in 2003 (Gendicine™). This treatment was based on an adenoviral gene delivery system that was capable of inserting the p53 gene into tumor cells, thereby stimulating cell death. Gendicide™ was approved for the treatment of head- and neck squamous cell carcinoma (14).

Regulation (EC) No. 1394/2007 was set up in Europe in 2007 but was only effective a couple of years later. In June 2009, ChondroCelect was the first product with a draft positive opinion by the CAT in relation to an initial marketing authorization. This cell-based medicinal product comprised of characterized viable autologous cartilage-forming cells expanded *in vivo*, expressing specific marker proteins, intended for the repair of single symptomatic cartilage defects of the femoral condyle of the knee, in adult patients (25).

In the meantime, in 2008, Cerepro^®^ became the first adenoviral vector to complete a phase III clinical trial (26). The treatment consisted in administering the herpes simplex virus gene for thymidine kinase (TK) encased in a non-replicating AdV vector, followed by administration of ganciclovir, in patients with operable, high-grade malignant glioma. Transduced cells express TK which phosphorylates ganciclovir that is further phosphorylated by several cellular kinases. The final product is ganciclovir triphosphate which is incorporated into DNA of dividing cells, as opposed to deoxyguanosine triphosphate, causing chain termination and apoptosis (27, 28). A MAA was submitted but the CHMP adopted a negative opinion in December 2009, and the company requested a reexamination of the opinion. During this period, in early 2010, the applicant requested withdrawal of the application on the basis that it had been unable to demonstrate to the Committee that its main study provided clear evidence of a clinically meaningful benefit in relation to risk (29).

Finally, in July 2012, the EMA recommended for the first time a gene therapy product (Glybera, alipogene tiparvovec) for approval in the European Union. Glybera is based on an adeno-associated viral (AAV) vector and gained approval for the treatment of a genetically inherited metabolic disorder related to the gene encoding the lipoprotein lipase (LPL). LPL is a key enzyme in the metabolism of lipoproteins following fat intake with diet. The lack of functional LPL results in severe hypertriglyceridemia, episodes of abdominal pain, acute pancreatitis and eruptive cutaneous xanthomatosis (30).

Glybera paved the way for the approval of other gene therapy products in Europe. Since then, Imlygic and Strimvelis were granted marketing authorization.

Amgen’s Imlygic (talimogene laherparepvec) is an oncolytic immunotherapy which uses an attenuated herpes simplex virus-1 (HSV-1) as a vector. Imlygic is indicated for the treatment of adults with unresectable melanoma that is regionally or distantly metastatic with no bone, brain, lung or other visceral disease (31, 32).

GSK’s Strimvelis (autologous CD34+ enriched cell fraction that contains CD34+ cells transduced with retroviral vector that encodes for the human ADA cDNA sequence) was the first *ex vivo* stem-cell gene therapy to be approved in Europe. The drug is meant to be used in patients with ADA-SCID who are not suitable to undergo bone-marrow transplant due to lack of matching donor (33, 34).

### Gene Delivery Vectors

Over the years, one of the most significant challenges of gene therapy has been the effective and safe delivery to its target. In light of the multiple extra and intracellular barriers gene delivery strategies came into picture, specifically through vehicles also known as vectors (2, 35).

The ideal gene delivery systems should have:

- high gene transfer efficiency
- low toxicity to the cells
- single cell specificity to the intended target
- the ability to simultaneously treat heterogeneous systems with many different cells (16).

Current non-viral gene delivery methods may be grouped into two different categories: physical or chemical. Physical gene delivery strategies use a wide variety of physical methods such as microinjection, needle injection, jet injection, gene gun/DNA injection/DNA-coated particle bombardment, electroporation, sonoporation, hydrodynamic gene transfer, and mechanical massage. On the other hand, examples of chemical gene delivery methods include calcium phosphate precipitation, cationic lipids (liposomes), cationic polymers, and lipopolyplexes (13, 15, 16).

When considering non-viral vectors, a number of advantages should be taken into consideration, such as easy scale up production, ability to carry large molecular size genes and lack of viral component, i.e., low immunogenicity. On the other hand, the high vulnerability to intra- and extracellular degradation, with subsequent low cellular uptake is a major drawback as well as the low transgene expression, i.e., low efficacy (15).

Viral vectors are based on removing the pathogenicity of specific virus to use them as carriers of the therapeutic genetic content. Some of the most frequently used viral vector families include AdV, AAV, herpes simplex virus (HSV), and retrovirus (such as gammaretrovirus and lentivirus). Main differences among these viral vectors are presented in Table 1 (36–39).

**Table 1:** Viral vectors overview.

Viral vector family	Immunogenicity	Genomic integration	Transgene expression	Packed genome size	Advanced therapy medicinal product examples
Adenovirus	High	Non-integrating	Transient	Intermediate	Advexin, Cerepro
Adeno-associated virus	Low	Non-integrating	Potentially long lasting	Low	Glybera
Herpes simplex virus (HSV)	High	Non-integrating	Potentially long lasting	Intermediate	Imlygic
Retrovirus (gammaretrovirus and lentivirus)	Low	Integrating	Long lasting	High	Strimvelis, Kymriah

Advantages of viral vectors include the high cellular uptake, the high transduction efficacy and long-term gene expression. By contrast, safety concerns including immunogenicity are considered major drawbacks. Choosing a vector with low immunogenicity such as AAV as opposed to AdV reduces the risk of severe unwanted immunologic responses. On the other hand, integrating vectors such as those based on lentivirus will pose a higher risk for oncogenicity, compared with, for instance, AAV. In addition, poor target cell specificity may be a concern. For instance, recombinant AAV’s tropism is largely dependent on the capsid. Capsids may be covered by signaling peptides or “shuffled” (pseudotyped) to generate new capsids (40). Finally, inability to transfer high molecular weight genes and high production costs represent significant disadvantages when considering these types of vectors to incorporate potential ATMPs (15).

#### Gene Therapy Strategies: From *In Vivo* Modification to *Ex Vivo* Gene Transfer

Essentially, gene therapy may be performed by one of two approaches. *In vivo* gene therapy consists of directly administering the vector carrying the therapeutic gene into the target tissue. It involves administration of the vector directly in the patient and genetic modification occurs in the host. Another alternative is *ex vivo* gene therapy, typically used in diseases where a specific type of cell is affected, it is possible to modify cells outside the body of a patient or donor to express specific genes. The first step is to isolate the target stem, progenitor or differentiated cells. Then, the cells are expanded with or without genetic modification. Lastly, the product is reinfused back to the patient (41).

When compared with *in vivo* gene therapies, there are two important advantages. On the one hand, this method prevents direct human exposure to the vector which, in theory, decreases its immunogenicity, contributing to stronger safety profile. On the other hand, it is possible to select the target cells of transduction, thus improving specificity and efficacy (41).

Ideally, easy to isolate and to manipulate *ex vivo* cells would be the perfect choice to apply this strategy. Hematopoietic stem cells (HSC) fit both criteria. In addition, a long-term therapeutic effect is expected to be obtained as HSC originate several cell types, such as red blood cells and major immune cells (2). In the early 2000s, in Italy, 10 children with SCID due to ADA deficiency were treated with HSC transduced with a retroviral vector, which successfully engrafted and differentiated into myeloid cells containing ADA gene (42). Another example, also from Italy, showed promising results after treating three children with Wiskott–Aldrich syndrome (WAS), an inherited immunodeficiency caused by mutations in the gene encoding a regulating cytoskeleton protein (WASP). Hematopoietic stem/progenitor cells of the patients were genetically modified using a lentiviral vector encoding the functional WASP gene. The children were reinfused with the corrected cells after reduced-intensity conditioning regimen (43).

Other cell types used in *ex vivo* gene therapy include T cells. An established cell and gene therapy application is adoptive immunotherapy, where T cells are modified to better act against malignancies, infections and autoimmune diseases (41). Multiple studies were carried out by expanding and genetically modifying this cell type, particularly in the treatment of some lymphoproliferative diseases. In acute lymphoblastic lymphoma (ALL) a specific type of B cells accumulates in the body. Lymphadenopathy impairs immunity, allows opportunistic infections, and may compress adjacent structures. In 30–50% of patients, the lymphoblasts infiltrate bone marrow, causing unsuccessful hematopoiesis. In ALL CD19+, the proportion of immature B cells expressing the CD19 marker is high. Chimeric antigen receptor (CAR) therapy represents a therapeutic alternative recently approved by the US FDA for a specific subset of patients, namely relapsed and refractory CD19 malignancies. Novartis’ Kymriah™ (tisagenlecleucel) consists of genetically modified autologous T cells expressing an Anti-CD19 CAR and it has shown great promise in several clinical trials, with complete remission (CR) rates ranging from 67 to 90% (44–48).

In 2006, Yamanaka and his team managed to reprogram differentiated cells into induced pluripotent stem cells (iPSC), by transducing skin fibroblasts with viral vectors carrying specific gene transcription factors. These factors were not randomly chosen but rather identified as key in the maintenance of pluripotency in both early embryos and embryonic stem cells. The development of iPSC technology was such an important milestone that Yamanaka was awarded with the Nobel Prize in Physiology/Medicine, in 2012 (49, 50). Combining *ex vivo* gene transfer with iPSC may have high potential for the treatment of a number of genetic disorders. For example, transducing iPSC with a functional copy of β-globin gene showed promising results both in the treatment of β-thalassemia whether in *in vitro* (51) and in *in vivo* models (52). However, further studies are needed on this topic as it has been shown that iPSC may implicate some unacceptable safety risks in clinical application. For example, the presence of reprogramming factors (such as c-Myc), could induce tumorigenesis (53, 54).

Other types of cells that may be used for *ex vivo* gene transfer and yielded positive results in the potential treatment of several diseases include, but are not limited to, epidermal and limbal stem cells, neural stem/progenitor cells, cardiac stem cells and multipotent stromal cells (41).

In *ex vivo* gene therapy, the goal is to permanently modify the host genome, and then expand the cells before reinfusion (2). Retroviral vectors are the preferred choice for *ex vivo* gene therapy, since these require proviral integration into the host genome for transduction, and generally infect only dividing cells. The use of lentiviral vectors, mostly derived from HIV, which have a stronger safety profile and also transduce non-dividing cells may be preferred over gammaretroviral vectors (2, 55).

An alternative option to viral vectors is applying targeted genome editing using clustered regularly interspaced short palindromic repeat (CRISPR)–CRISPR-associated (CRISPR–Cas) systems. The potential for gene editing associated with the CRISPR/Cas9 technology was developed in the US by Doudna and Charpentier. It generally consists of cutting genomic DNA in a sequence-specific fashion, allowing for disruption or repair of that region. The greatest advantage of this method over using viral vectors is related to the low risk of immunogenicity but also low probability of insertional mutagenesis (IM) (2, 56). The most significant limitation of CRISPR/Cas9 is related to off-target mutations, which is discussed in further detail in a later section of this review.

DNA Transposition is a process by which discrete DNA portions, called DNA transposons, change their positions within the genome *via* a “cut and paste” mechanism. The process is mediated by the transposase enzyme that is responsible for removing the element from its donor plasmid, followed by reintegration of the transposon into a specific chromosomal site. Transient transfection of a transposase, together with a donor plasmid containing the gene of interest can also be a strategy for *ex vivo* gene transfer (41, 57).

### Strategic Clinical Applications

#### Monogenic Diseases

Most of the investigation in gene therapy is focused on monogenic diseases, as these are perfectly characterized through a defective single gene, making gene replacement a straightforward strategy. In addition, appropriate non-clinical animal models are relatively easy to obtain (35).

##### LPL Deficiency and Glybera

Lipoprotein lipase deficiency is a rare monogenic autosomal-recessive disease caused by a mutation in the gene encoding the LPL enzyme. LPL enzyme is involved in the fatty acids metabolism, by breaking them down into smaller molecules and allowing subsequent gastrointestinal absorption. As a result, LPL-deficient (LPLD) patients have an absence in the enzyme’s activity and are restricted to a low-fat diet, suffering from recurrent life threatening pancreatitis. Therapeutic management of LPLD is mostly based on strict adherence to a low-fat diet. However, compliance with such a diet is variable and difficult (30).

Glybera, the first GTMP approved by the EMA, in 2012, consists of a recombinant adeno-associated serotype 1 vector (rAAV) containing a functional copy of the LPL human gene. The drug administration is dependent on the patient’s weight and requires some level of anesthesia, since it involves several intramuscular injections. The gene is transduced within myocytes and results in production of LPL to compensate the loss-of-function, in such a way that the vector in unable to reproduce itself.

As an orphan medicine, Glybera was evaluated by the regulators with limited clinical data in a very small number of patients. The clinical development program included three open label uncontrolled studies, which treated an overall number of 27 patients. The process underwent two reevaluations before final approval. In terms of safety, most of adverse reactions are local and self-limiting within few days after the treatment. The risks associated with Glybera include significant tissue swelling caused by multiple injections and subsequent thrombogenicity, and risks associated with 3-month course of immunosuppression (recommended after drug administration) (58).

The primary efficacy endpoint presented in the submission package consisted on the reduction of serum triglycerides. However, this was not consistently achieved and, when it was observed, it was not sustained. Further analysis concluded that serum triglycerides were simply too variable in these patients, requiring the applicant to propose a new primary endpoint. The measurement of postprandial serum chylomicrons before and after gene therapy made biological sense. The data were compelling in the few subjects in which it was measured (8, 58).

##### Severe Combined Immunodeficiency (SCID) and Strimvelis

One of the clinical applications of *ex vivo* gene therapy is to reconstitute dysfunctional cell lineages and this can be accomplished by genetic replacement, for example, in the treatment of SCID using HSC that undergo *ex vivo* modification.

Combined immunodeficiencies (CID) comprise a heterogeneous group of genetic disorders that result in impaired development, function, or both of T lymphocytes, associated with a defective antibody response. In the most severe forms of CID, also known as SCID, there are practically no functioning peripheral T cells (20, 21, 41).

Just about half of all SCID cases are due to a defective development of T cells and NK cells as a result of mutations in the gene encoding interleukin 2 receptor-γ (IL2RG). This is called X-linked SCID, as it is related to a mutation in the X-chromosome. It is also generally known as the “Bubble Boy Disease,” named after a case in the late 70s of a young boy who lived over 10 years in a protective sterile plastic bubble, and then unfortunately died after an ineffective bone marrow transplant (59). Full activation of the IL2RG results in T-cell proliferation, antigen-induced cell death and boosting of cytolytic activity of NK cells. This mechanism is significantly impaired in patients with X-linked SCID (20, 60, 61).

Another highly common type of SCID is ADA-SCID, where a deficiency in adenosine deaminase is found. The lack of ADA enzyme results in (de)adenosine compounds accumulation, which in turn induce cell death, particularly of lymphoid progenitors. Patients with ADA-SCID have nearly full absence of lymphocytes, either T, B, or NK cells (20).

For both X-linked and ADA-SCID, hematopoietic stem cell transplantation (HSCT) represents life-saving standard of care therapy. The clinical prognosis in primary immunodeficiencies after HSCT is influenced by multiple factors, including molecular defect, disease status, donors, stem cell source and chemotherapy conditioning regimen. Conditioning aims at creating space in the recipient marrow enabling donor stem cells to engraft more easily (62). Risks include infection during the transplant period, as patients undergo strong immunosuppressant regimen, as well as development of acute and/or chronic graft-versus-host disease (GvHD). GvHD occurs in allogenic transplants where newly transplanted cells attack the transplant recipient’s body. Here, gene therapy represents a significant advantage as the patient’s own cells are modified and reinfused back into the patient. This means that the donor receives his/her own cells (autologous transplant). GvHD is less likely to occur with human leukocyte antigen (HLA)-matching donor (63, 64).

In the early 1990s, Blaese and his team were first to conduct a trial using a therapeutic gene, by treating children with ADA-SCID (19). It was not until 2016 that a GTMP was authorized to treat ADA-SCID. Strimvelis comprised of patient’s own CD34+ enriched cell fraction containing CD34+ cells transduced with retroviral vector that encodes for the human ADA cDNA sequence.

Strimvelis’ intends to treat ADA-SCID patients who cannot undergo bone marrow transplant as they have no suitable donor. Before treatment administration, a conditioning regimen with busulfan is required, after bone marrow collection. The patients are then given transduced autologous cells *via* intravenous administration (33).

As far as manufacturing, Strimvelis requires particular cell processing capabilities, in a short-time frame, taking into account the cells viability. This process takes place in Italy (Molmed) which currently is the only approved manufacturing site. The patients are expected to travel to Italy to receive treatment (33).

As for Glybera, Strimvelis is proposed as a one-time administration to address an orphan disease. The pivotal study included a very limited number of patients (12 subjects). In terms of efficacy, and considering that ADA-SCID is a fatal disease where patient do not survive over the first year of life, the EMA considered that there was compelling evidence of benefit. Indeed, all patients were alive after a median follow-up of 7 years (33, 65).

Immune reconstitution appears to be much slower with gene therapy when compared with HSCT. Therefore, the risk related to infections was considered high by the EMA, especially during the first year after the treatment. Autoimmune serious adverse events were noted namely hemolytic anemia, aplastic anemia, hepatitis, thrombocytopenia, and Guillain–Barré syndrome. However, considering the strong efficacy data, the risk benefic balance was positive, as per the regulator’s assessment (33).

##### Hemophilia B and scAAV2/8-LP1-hFIXco

Hemophilia B is a severe inherited blood disorder caused by a deficiency in the gene encoding human clotting factor IX (FIX). As a result of this loss-of-function, patients with hemophilia have low levels of FIX, and a high risk of spontaneous bleeding while performing daily activities. A specific group of patients shows a severe bleeding phenotype which results in spontaneous musculoskeletal and soft tissue hemorrhages in the absence of appropriate treatment (66, 67).

Intravenous administration of recombinant clotting factor concentrates represents the standard of care therapy. Due to its relatively short half-life, patients need to be administered rather frequently, around 2–3 times a week. PEGylated clotting factors may resolve this issue to a certain extent, by allowing treatment every 2 weeks. However, this is still not a curative approach and the risks of lifelong administration of PEGylated proteins are not completely known (66, 67).

The first hemophilia gene therapy studies used AAV2 as a vector and different routs of administration. Intramuscular injection of AAV2-FIX in a group of eight patients, showed no significant safety concerns though limited efficacy was observed, likely related to levels of FIX not rising above 1%. Conversely, improved efficacy was seen in a trial where seven patients received FIX encapsulated in AAV2 vector administered directly in the hepatic artery. However, some safety issues related to immunogenicity toward the viral capsid were noted. In addition, preexistence of neutralizing antibodies (Nab) could potentially impact successful gene transduction (68, 69).

A group of London based investigators decided to use a different AAV serotype and a more straightforward route of administration. Early phase I dose-escalation trial with 10 patients using a self complementary AAV serotype 8 vector expressing codon-optimized human FIX under the control of a liver specific promoter (scAAV2/8-LP1-hFIXco) have shown promising results, following a single systemic administration of the vector in severe hemophilia adult patients. AAV8 has an outstanding tropism for hepatic cells which is ideal as the synthesis of the defective clotting factor takes place in the liver.

There was an evident analytic increase in plasma FIX activity (less than 1 to 1–6%) and from a clinical perspective the average annual number of bleeding episodes was consistently lower after gene transfer, particularly in patients in the high-dose cohort. From a safety perspective, there were a number of cases of asymptomatic, transient elevation of serum liver enzymes, probably as a result of a cellular immune response to the AAV8 capsid, which rapidly disappeared after prednisolone treatment (70).

##### Cystic Fibrosis (CF) and pGM169/GL67A

Cystic fibrosis is an autosomal recessive disorder which impacts the protein encoded by the cystic fibrosis transmembrane conductance regulator (CFTR) gene. The CFTR protein is present in epithelial membrane cells, widely distributed throughout the body, including in the pulmonary tract and gastrointestinal tract. Loss-of-function of the CFTR gene leads to intracellular accumulation of chloride, sodium, and water which is of particular severity in the lungs, since it leads to formation of a thick mucus layer, impairing ciliary clearance pathway and being a perfect breeding media for microorganisms. Subsequent accumulation of inflammatory cells and other mediators may lead to bronchiectasis and gradually, overtime, airway remodeling takes place and the airway is destroyed (fibrosis). In late stages, CF leads to respiratory failure and chronic lung infection which is the main responsible for morbidity and mortality (71, 72).

Therapeutic management of CF, especially displaying pulmonary exacerbations, is mainly based on administration of inhaled bronchodilators, mucolytic agents and use of oral antibiotics.

Epithelial respiratory cells are an attractive target which provide easy access when compared with other gene therapy strategies requiring more invasive forms of administration such as intramuscular or intravenous injection. Attempts to treat CF have been reported using both viral (73) and non-viral vectors (74) carrying the gene encoding the functional CFTR protein.

Repeated nebulization of plasmid DNA encoding the CFTR gene complexed within a cationic liposome (pGM169/GL67A) was tested in CF patients. This phase 2-b trial enrolled 140 patients and showed proof-of-concept that non-viral gene therapy could beneficially impact lung function in CF patients. Treatment was well tolerated and a significant though modest effect was seen in the forced expiratory value in 1 s versus placebo after 12 months of treatment (74).

Dose increase or shortening of the administration interval were considered as an improvement strategy. On the other hand, more potent vectors like viral vectors were also tested in CF animal models. Lentiviral vectors have been investigated but since these vectors lack a natural tropism for lung tissue, pseudotyping with envelope proteins is required for the viral particles to reach their target. Promising results including a transduction of the gene in the respiratory epithelium of the murine nose *in vivo* at levels that may be relevant for clinical benefit in CF patients were reported by capsid pseudotyping with hemaglutinin-neuraminidase (HN) proteins from Sendai virus (73).

#### Multifactorial Diseases

As opposed to monogenic disorders, other more complex diseases may also be a suitable target for gene therapy. Here, gene replacement might not be the most suitable choice as for monogenic diseases. Conversely, gene addition in combination with other therapeutic agents has been studied in specific diseases and yielded interesting results.

##### Heart Failure (HF) and AAV1/SERCA2a

Heart failure is a clinical syndrome where, generally, the heart fails to pump sufficient blood to meet the body’s metabolic needs, as a result of a decrease in cardiac function. Underlying HF causes include post-acute myocardial infarction status. HF is characterized by shortness of breath, swollen ankles and fatigue and may be accompanied by signs such as elevated jugular venous pressure, pulmonary crackles and peripheral edema (75).

Current therapeutic management in an outpatient basis consists of oral angiotensin-converting enzyme inhibitor, beta-blockers and mineralocorticoid/aldosterone receptor antagonist. However, HF has an overall prevalence that is increasing globally and, therefore, represents a major public health issue characterized by significant mortality, frequent hospitalization and poor quality of life (75).

Calcium is one of the most important ions involved in cardiac function and contractility. Deficient uptake of cytosolic calcium to the sarcoplasmatic reticulum has been identified in cardiac cells from failing human hearts. The enzyme involved in this process (the sarcoplasmic reticulum Ca-ATPase, also referred to as SERCA2a) was noted to have a reduced expression and activity in HF, not necessarily due to a defect in the corresponding genes (35, 76).

The pilot dose-finding phase II CUPID study was the first human trial with gene transfer of SERCA2a. This was a small, placebo-controlled study in advanced HF patients which tested the percutaneous administration of a SERCA2a gene encapsulated in an AAV serotype 1 vector on symptomatic, functional and structural efficacy endpoints. 39 Patients were on optimal medical treatment in addition of being administered with the vector directly in the coronary circulation and results were very positive, without any significant safety concerns (77, 78).

However, a larger phase IIb trial in 250 patients (CUPID 2), which tested the same vector in a broader patient population, showed no evidence of improved outcomes at the studied dose. This did not support the same encouraging results as the pivotal trial. Investigators provided several justifications including that the results of the pivotal trial were consequence of a chance finding and that the patients randomized to the placebo arm, in the CUPID trial, had a greater severity of illness. Another potential reason was related to the proportion of empty viral particles administered to the trial subjects that was higher in the CUPID trial when compared with the CUPID 2. These empty particles may improve transduction of the vector by binding to self-antibodies against the vector (79).

##### HIV Infection and Vectored Immunoprophylaxis (VIP)

Currently, HIV has no curative therapy though patients are able to live for many years while still infected if appropriate antiretroviral treatment (ART) is administered. ARTs suppress viral replication to low or undetectable levels, with a corresponding but variable increase in CD4 T-cell counts. Even though HIV infection has become a chronic but manageable disease, a significant decrease in survival is observed as a result of long-term complications in main organ systems such as accelerated cardiovascular disease, liver and renal failure and neurocognitive dysfunction. In addition, resistance to certain ARTs suggests that further alternatives should be investigated (80).

A large number of attempts have been made at testing not only new treatment options but also preventative strategies, such as the development of vaccines. Here, the discovery of broadly Nab represents an important milestone. Natural infection induces the production of non-neutralizing or strain specific antibodies, especially during the early months after infection. Broadly Nab are antibodies against several strains of HIV type 1 (HIV-1) and can be found in approximately 20% of HIV-1 infected patients (81).

Intramuscular delivery of adeno-associated virus containing a gene encoding broadly Nab against Human or Simian immunodeficiency virus has been tested in both rodent (82) and non-rodent animal models (83), with encouraging results. This strategy is also called VIP, and efforts are currently underway for extending this strategy to humans, for the first time.

##### Cancer, Kymriah, and Imlygic

Cancer is a complex disorder where generally multiple genes are affected. In addition, substantial differences can be found between tumor of different individuals and between tumors in the same patient. Gene addition as cancer treatment is not as straightforward as in monogenic diseases. Two important strategies are worth mentioning, one of them is already approved by the US FDA and the other by the EMA (84, 85).

One the one hand, in 2017, Kymriah (tisagenlecleucel, an *ex vivo* genetically modified T-cells to express the anti-CD19 CAR) was the first product based on gene therapy approved by the US FDA. Positive results were shown in relapsed and refractory ALL patients. Here, a lentiviral vector containing the gene encoding the CAR-19 gene is transduced in patients own T-cells and then reinfused back unto the patient’s circulation (44, 45, 48, 85).

On the other hand, in 2016, Imlygic (talimogene laherparepvec) was the second gene therapy product approved in the EU, which takes advantage of a gene addition strategy for the treatment of advanced unresectable melanoma. Herpes simplex vector is administered directly into the tumor. This vector was subjected to specific viral gene deletions, which result in replication inside tumor cells and consequent oncolysis. Furthermore, the vector contains a gene encoding the granulocyte macrophage colony-stimulating factor (GM-CSF), which triggers a systemic immune response, capable of fighting not only the injected tumor but also its metastasis. The main phase III trial that supported the MAA was based on a comparison between patients treated with subcutaneous GM-CSF versus Imlygic. The study showed that the investigational treatment significantly improved the rate of responses lasting continuously for 6 or more months in patients with unresected stage IIIB to IV melanoma compared with subcutaneous GM-CSF. Imlygic’s safety profile was considered acceptable, inducing minor adverse reactions mainly related to flu-like syndrome, following intralesional administration (31, 86).

#### DNA Downregulation through RNA Targeting

RNAi works by suppressing the expression of certain mRNAs, thereby preventing the accumulation of the corresponding toxic protein. Even though there are currently no approved ATMPs based on this strategy, silencing a toxic gene may bring therapeutic benefit in specific genetic disorders.

##### HIV Infection and Small Hairpin RNA (shRNA) against CCR5 Gene

Virtually all HIV target cells are produced from HSC, including T cells, macrophages, dendritic cells and brain microglia. Here, the virus is permanently incorporated forming “reservoirs” of infected cells that are unable to be eliminated. The outstanding case of the “Berlin Patient” raised great hope toward uncovering a cure for HIV. In 2007, an HIV infected patient was treated for relapsed acute myeloid leukemia with HSCT. This resulted in the first documented case of HIV cure, highlighting the importance of the chemokine receptor 5 (CCR5) in maintaining HIV infection. The transplanted cells had the CCR5 gene naturally silenced since the donor was homozygous for a deletion in the CCR5 gene providing resistance against HIV-1 infection. From a molecular perspective, cellular infection with HIV-1 requires a CD4+ cell and a CCR5 receptor and by disabling the CCR gene the virus is unable to infect body cells. Up until today, the patient remained free of leukemia and also free of HIV rebound after discontinuing ART. However, this is not a feasible treatment option for the majority of HIV patients, since it would be very difficult to find an HLA-matching donor who would simultaneously be HIV-resistant by displaying the required CCR5 homozygous deletion (87).

By contrast, the “Berlin Patient” results were key for other gene therapy investigators to test administration of vectors containing anti-HIV genes. For example, in an attempt to knock down the CCR5 gene, several groups tested the administration of shRNA against CCR5 encapsulated within a lentiviral vector (88). shRNAs are vector-derived RNA interference structured, ultimately processed to produce siRNAs in the target cells (89).

The *in vitro* results showed that the cells gained HIV resistance. However, overexpression of shRNA could induce cytotoxicity in human primary T lymphocytes. In an optimized animal model, no apparent adverse effects due to the shRNA were evident in transplanted primates for 3 years (88).

##### Paramyloidosis and Patisiran

A similar strategy was used by a group of investigators, in the treatment of transthyretin amyloidosis. This is a dominant autosomal disease where hepatocyte-derived transthyretin amyloid deposits accumulate in several tissues and organs, namely, peripheral nerves and in the gastrointestinal tract, heart and kidneys. The signs and symptoms include pain, paresthesia, muscular weakness, and autonomic dysfunction.

Tafamidis, a small-molecule stabilizer of the transthyretin tetramer, is the only approved treatment, slowing the progression of neuropathy. Hepatic transplant eliminates the production of mutant transthyretin though there are obvious limitations regarding the broad application of this therapeutic option, such as HLA compatibility issues.

Patisiran is an antitransthyretin small interfering RNA encapsulated in lipid nanoparticles that was tested in both rodents and humans. Clinical results showed that Patisiran suppressed the production of both mutant and non-mutant forms of transthyretin, which may lead to an improvement of disease related symptoms. Besides infusion-related adverse reactions, the preliminary data on safety were satisfactory. A phase III study is currently ongoing to establish efficacy and safety of the investigational medicinal product (90, 91).

One potential challenge associated with RNA interference particularly impacts dominant genetic diseases, where there is one mutated allele and one normal allele. Here, RNAi inhibits the production of both the mutated and the normal protein, which can lead to a decline in the gene’s normal function. A possible strategy to overcome this hurdle may include the administration of allele-specific RNAi toward the mutated allele, which has been tested by some investigators in some pathologies such as Huntington’s disease (92).

#### Targeted Gene Editing

The greatest advantage of targeted gene editing when compared with gene replacement or addition is the highest control over the defective gene. Theoretically, it corrects the problem directly in the source, rather than adding another genetic sequence. As simple as it may appear, targeting a single gene within a large genome may be challenging. This is probably the strategy that is being developed with the most caution due to potential important safety events, such as off-target effects and also ethical implications about possible genetic changes in germline cells.

Three important strategies should be addressed including zinc finger nucleases (ZFN), Transcription activator-like effector nucleases (TALENs) and CRISPR–Cas (35, 93).

##### HIV Treatment *via* CCR5 Gene Editing Using ZFN

ZFN were the first genome editing nucleases to be described and are a type of gene-targeting reactants which combine both DNA recognition specificity of ZFN and the enzymatic activity of *Fok*I. The zinc finger domain comprises 30 amino acids and coordinates one zinc atom using two histidine and two cysteine residues. A specific DNA triplet is recognized by an α-helix in each domain. Multiple zinc finger domains are able to recognize long DNA sequences. *Fok*I is a nuclease responsible for the double-stranded break of DNA. The nucleases attached to ZFNs are required to function as dimmers, which mean that ZFNs can target any specific DNA sequence.

After this targeted cleavage, two DNA repair mechanisms can take place, including homologous recombination or non-homologous end joining. Homologous recombination repairs the break while maintaining the original DNA sequence. This can be used for targeted gene replacement. Non-homologous end joining can be used to edit a specific gene as it may result in deletion of a specific DNA sequence at the break site, causing permanent disruption of the primary DNA sequence (94).

The first clinical trial using a nuclease for targeted gene editing (93) was conducted in 12 HIV patients where the CCR5 gene was silenced by treatment of patients’ own CD4 T cells with ZFN. In this phase I study the patient’s own cells were treated *ex vivo* with ZFN to achieve CCR5 gene disruption and reinfused back into circulation. The study results included a significant increase in CD4 T cells count after administration and long-term persistence of CCR5-modified CD4 T cells in peripheral blood and other tissues. Overall, the results showed that artificial induction of HIV resistance was a generally safe and feasible approach (95).

##### Leukemia and CAR 19 T Cells Developed with TALENs

Transcription activator-like effector nucleases have rapidly became an alternative genome editing tool to ZFN. The non-specific *Fok*I domain is used as the DNA cleavage element inducing double strand breaks. The DNA binding domains comprise a series of tandem repeats, each including around 33–35 amino acids capable of recognizing a single nucleotide. TALEN–DNA interactions are less complex when compared with ZFN. In addition, designing TALENs is generally simpler than ZFN. The bulky size of TALENs might be a limitation in clinical application (96, 97).

The first published clinical application of TALEN refers to treatment of an 11-month old baby with B acute lymphoblastic leukemia (B-ALL). Phase I trials for this specific GTMP were underway though the research group received a request for therapy on a compassionate basis for this infant with refractory relapsed B-ALL. Under UK special therapy regulations, this was the first patient treated with TALEN engineered CAR 19 T Cells. Analysis of the short follow-up period, the intervention that included lymphodepletion and infusion of the manipulated CART19 T cells has induced molecular remission where previous conventional treatments had failed (98).

##### Immunesuppression and CRISPR–Cas9

Clustered regularly interspaced short palindromic repeat technology allows gene editing with unprecedented accuracy and the potential to become a powerful gene editing tool was found by accident through a project on characterization of CRISPR-associated protein 9 (Cas9 enzyme) by Doudna and Charpentier.

The term CRISPR refers to specific DNA sequences initially found in bacteria DNA as a series of short direct repeats interspaced with short sequences. The role of these sequences is related to protection from viral and plasmid infection. CRISPR DNA sequences within the host cell are specific for each virus. Transcription of this DNA to RNA is used to recognize a new virus attack. Together with a second small RNA, tracrRNA (trans activating crRNA), a Cas enzyme is able to recognize and neutralize viral DNA, preventing the infection.

Doudna envisioned that it would be possible for Cas9 to target a specific DNA sequence, by using a defined RNA template coupled to the enzyme so that it acts on the desired gene (56, 99).

A group of Chinese investigators have generated genetically modified rodents and non-human primates by effectively disrupting specific genes, through the CRISPR–Cas9 technology in embryonic cells (100, 101). This technology is on the verge of being tested for the first time in humans, by *ex vivo* removal of the programmed cell death protein 1 (PDCD-1) gene in T cells.

Programmed cell death protein 1 is a key immune checkpoint receptor expressed by activated T cells and it is responsible for immunosuppression. Immunosuppressive PDCD-1 ligands are expressed by a number of tumor cells. Therefore, inhibition of this receptor may enhance T-cell response. Nivolumab is a monoclonal antibody, currently approved by the EMA, for the treatment of an array of cancer types such as melanoma, non-small cell lung cancer and renal cell carcinoma (102).

The same group of Chinese investigators is behind the first human trial involving the CRISPR–Cas9 technology in disrupting the PDCD-1 gene. To date, data from www.clinicaltrials.gov display four planned first-in-human studies through the *ex vivo* modification of T cells so that the PDCD-1 gene is knocked out using CRISPR–Cas9. These cells are then reinfused back into patients’ own circulation. The group has seen that the strategy is promising *in vitro*, by first applying it to human T cells from cancer patients (103, 104).

Recently, CRISPR–Cas9 made headlines again as a group of US investigators used the technique for the first time in viable human embryos to correct an inherited genetic mutation. Patients with an autosomal dominant genetic condition affecting the *MYBPC3* gene may develop hypertrophic cardiomyopathy. This is a disease characterized by, among other clinical features, left ventricular hypertrophy. The tested embryos were not meant for implantation. Even though none of the embryos developed for more than a few days, the results were promising as not only the genetic mutation was corrected but two important safety issues seemed to be addressed. On the one hand, from the 58 tested embryos, only one showed signs of mosaicism. This is when in a single cell with different genetic sequence is found in the same embryo, which is unacceptable since it would make preimplantation genetic diagnosis challenging. Finally, there was no evidence of off-target mutations (105).

While the scientific community is excited about this technology and the expectation are high for these first-in-human studies, some limitations have been reported for CRISPR technology. Off-target mutations detected in higher proportions versus the intended gene edition are likely to occur and are a major concern in clinical application. Several strategies, at the molecular level, to decrease the off-target mutations have been developed, as well as new approaches to detect them (106).

### The Challenges

When comparing to classic chemical or biologic therapies, ATMPs are substantially different in nature and, consequently, the evaluation of a MAA may not follow the same “standardized” data submission package. In Europe, the EMA has developed a document outlining a risk-based approach for the evaluation of these specific medicinal products. The “risk-based approach” is defined as “*a strategy aiming to determine the extent of quality, nonclinical and clinical data to be included in the MAA, in accordance with the scientific guidelines relating to the quality, safety and efficacy of medicinal products and to justify any deviation from the technical requirements as defined in Annex I, part IV of Directive 2001/83/EC*” (107). This is an optional approach that highlights some intrinsic risks as well as risk factors associated with candidate ATMPs. Interestingly, some of the risks and risk factors mentioned in this guideline are compatible with a number of preidentified challenges in ATMP drug development. In this section, these and other challenges will be discussed as well as potential overcoming strategies.

#### Safety Issues

##### Potential Immunogenicity

Patients who suffer from ornithine transcarbamylase deficiency have a rare X-linked genetic disorder characterized by complete or partial lack of the enzyme OTC. This is an enzyme involved in the urea cycle which prevents excessive accumulation of nitrogen, in the form of ammonia. Hyperammonemia may lead to neurotoxicity and, in extreme cases, result in coma and death.

In 1997, at the University of Pennsylvania, a group of investigators developed an AdV vector that contained a functional copy of the OTC gene. Eighteen patients with OTCD were enrolled in a phase I dose escalating study, which testes six different investigational product doses. The vector was administered through a femoral catheter into the right hepatic artery. In 1999, Jessie Gelsinger was enrolled and allocated to the highest dose cohort. Just 4 days after administration, a strong immune response against the vector was noted and the patient died due to multiorgan failure (24).

Following FDA inspection, the case unraveled major deficiencies in trial conduct, such as failure to report significant safety information to regulatory bodies, inadequate informed consent process, inclusion of ineligible patients and protocol amendment implementation before IRB approval. In addition, researchers’ financial interest in positive trial results was pointed out as potential bias (108–110).

In return of such concerns, the US government agencies and academic institutions strengthened regulatory requirements on clinical research with special additional requirements placed on clinical gene therapy trials. For instance, at the time, it became mandatory for early phase studies to have Drug Safety and Monitoring Boards (109).

Initial IND included non-clinical data from the first-generation vector in mice and rhesus macaques. At the highest dose, syndrome of severe liver damage was noted in monkeys, which lead to death. However, in light of further scientific advancements between initial IND and trial approval, a third generation vector was used in clinical trials. Improved toxicity profile was seen in mice and baboons, compared with the first generation. Therefore, patients in the high-dose cohort were administered with vector dose that was 17-fold lower compared with the dose of first-generation vector that showed severe toxicity in primates. Researchers estimated that this would provide a 100- to 1,000-fold margin of safety in terms of vector dose (108). Holistically, one can argue that the immunogenic profile of the vector was insufficiently characterized from a non-clinical standpoint, as well as that the researchers used potentially inadequate animal models. These data did not allow accurate prediction of the patient’s massive immune response reaction.

Both the viral vector and the transgene product may exert these reactions. The unpredictability of innate and antigen-dependent immune responses in humans is a huge barrier. In addition, suitable animal models to replicate these responses are difficult to be established (55).

Innate immunity is the first line human immune response, which is activated rather quickly after gene therapy administration. In a viral vector, capsid proteins as well as viral gene products may be recognized by the immune system as pathogens. When using non-viral vectors, naked DNA from plasmids may also exert innate and adaptive immune response. These have a higher proportion of unmethylated CpG motifs which have immunostimulatory effects (36).

Some vectors are more prone to induce unwanted immunogenic responses stressing the importance of choosing an appropriate vector type. During the manufacturing process, some vectors are more easily purified than others resulting in impurities in the finished product that may lead to immunogenic reactions. In addition, the biodistribution to non-target sites that are more immunogenic may be a source of concern. Since antibodies have limited access to specific body areas, stronger neutralization may occur after intra-hepatic or respiratory administration (where antibodies can more easily access) when compared with intraocular (retina) or intracranial (brain) administration. Moreover, immunity varies with medical procedure-related factors (e.g., locally administered high dose may cause site inflammatory response due to immune reaction to a therapeutic protein), patient-related factors (e.g., genetic background) and type of transgene and transgene expression levels after administration (e.g., existence of DNA promoters within the therapeutic gene). The latter are of particular importance especially the cytokines present at the site of transgene expression. These may influence inhibition or activation of promoters and, consequently, impact the expression of the gene of interest (36, 107).

Administration of immunosuppressive agents prior or after gene therapy exposure may prevent immunogenicity. Modification of the vector structure at the capsid proteins level or by eliminating viral genes may also be an appealing option. The antigen of the vector may be changed and no longer display the immunogenic effect (36). The AdV vector is known to be highly immunogenic and the use of other types of less immunogenic vectors such as viral AAV or other non-viral vectors may be a strategy to overcome this issue (55). In addition, *ex vivo* administration of gene therapy as opposed to *in vivo* delivery may also have the potential to exert less immunogenic responses (41).

##### Oncogenicity

Unwanted tumor formation may be a result of IM, which occurs when a gene vector integrates into the host genome, as a consequence of activation/upregulation of oncogenes or inactivation or downregulation of tumor/suppressing genes.

In 2002, a trial lead by Salima Hacein-Bey-Abina, in France, was the first to test *ex vivo* gene modification in patients with X-linked SCID. Five children underwent bone marrow harvesting and the CD34+ cells were then modified using a retroviral vector to express the gene encoding the common gamma chain (γc). Even though the transduction process had limited efficiency, the immune system of the 5 patients was partially repaired. At the time, these were very encouraging results and, In addition, no significant safety events were noted during the 30 month follow-up period (111). Later in 2004, the second X-linked SCID trial took place in the UK, enrolling four pediatric patients. Gene therapy strategy was very similar to the previously used by Hacein-Bey-Abina’s team though the viral vector was pseudotyped. Patients were followed for 29 months displaying a substantial clinical and immunological benefit. On the other hand, no serious adverse events were noted, at that point (112).

Between late 2002 and beginning of 2003, reports that two of the French patients developed leukemia alarmed the scientific community and the regulators. As a result, French Health Authority immediately suspended SCID gene therapy trials (113, 114).

The underlying cause was potentially related to the enhancer activity of the viral long-terminal repeat which activated an oncogene. The LMO2 (LIM domain only-2) is a cysteine rich *Lin*-11 *Isl*-1 *Mec*-3 (LIM) protein required for normal hematopoiesis. Retroviral integration in the proximity of the LMO2 proto-oncogene promoter resulted in abnormal transcription and expression of LMO2 triggered malignant cell proliferation. Since the two leukemia patients were the youngest and those who received the highest cell dose, these were identified as putative contributing risk factor (20, 115). It was not until June 2004 that the temporary halt was lifted. The HA required a protocol amendment to restrict the age of the patient population as well as to limit a maximum number of cells to be administered (116).

Over the next few years, in total, reports of leukemia were noted for four of the nine patients. Unfortunately, in October 2004, one of the patients died. These events highlighted the importance of adequate assessment of IM risk in gene therapy and, currently, in Europe, when submitting a MAA, Sponsors are expected to have data on IM for those candidate GTMPs which have that potential. Minimization of the risk of IM could be at the level of appropriate genetic regulation. In the X-linked SCID case, a potentially safer vector could be engineered based on removing the LTR enhancer element and adding an internal promoter which would modulate the properties of the preintegration factor. Another potential strategy could be directing the integration into neutral region of the genome (“safe harbor”) (20, 93, 117, 118).

Insertion profile as well as vector persistence of the vector should also be considered (107). Vectors that do not efficiently integrate into the host genome include AAVs, plasmids, or retroviral vectors modified to avoid integrations. Instead, the use of integrating vectors such as gammaretroviruses, lentivirus, and transposons may increase the potential for oncogenesis (119). However, compared with gammaretroviruses, lentiviruses such as HIV-1 are more likely to integrate within active transcription units not related to proliferation-associated genes or transcriptional start sites, which suggests a lower potential for triggering oncogenic adverse events (118).

Higher vector dose administration may have an increased potential for IM, as the number of integrations/transduced cells is directly proportional to the number of vectors present. In addition, the mechanism of action of the transgene product may also influence potential mutations. For example, if this product is involved in cellular growth then accelerated occurrence of mutagenesis may be observed.

Finally, the target cell population/organ of the GTMP is highly likely to influence the oncogenic profile. Generally, the risk of oncogenic events appears to be inversely related to the maturity of cells/tissues. For instance, gammaretroviral vectors can induce oncogenic events in HSC but not in mature lymphocytes, likely as a result of the different genetic program of the two cells types (119).

Several strategies were developed to evaluate the oncogenicity of GTMPs. Non-clinical integration studies are required for drug candidates that are expected to have IM potential. Moving on to the clinical studies, the oncogenic profile of a gene therapy product is difficult to be predict considering the limited experience in humans with a low number of patients that have been treated with vector to date, the longer follow-up periods that are required and the possibility that the baseline disease could contribute to increase the risk (120).

Strategies to overcome potential oncogenicity include modification of vector design to prevent activation of oncogenic genes at the integration sites, utilization of non-integrating vectors or highly targeted genomic integration at the desired chromosomal loci (121).

Considering these challenges and the often irreversible effects of gene transfer, the CHMP Gene Therapy Working Party developed a range of scientific guidelines to minimize these risks (121). The safety follow-up requirements for patients administered with GTMPs is one of the most important documents (122), detailing recommendations for clinical monitoring and safety follow-up to detect early or delayed signals of adverse reactions, prevent clinical consequences of such reactions, ensure timely treatment and gain insights on long-term safety and efficacy. The clinical follow-up activities described in this guideline should not be established in isolation but rather as an addition to the common pharmacovigilance requirements. Safety monitoring may be required within days, weeks or even years after gene therapy treatment administration. For example, an adverse reaction related to immunogenicity may be detected just a few hours after treatment administration, as opposed to an oncogenic safety event which may take years to be noted. Most of the recommendations for the different GT products include follow-up at pretreatment, 3, 6, and 12 months and then yearly thereafter for 5 years or longer. The decision on the extent and duration of clinical follow-up requires a case-by-case analysis since there are many different factors that should be taken into consideration (Table 2).

**Table 2:** EMA guideline on safety follow-up.

Factors that influence extent and duration of gene therapy clinical follow-up
Potential for and extent of chromosomal integration of a vector/gene Capacity of a vector/gene for latency/reactivation Capacity of a vector for inadvertent replication after complementation by viruses causing escape from latency and reactivation and eventually leading to mobilization Persistence of expression of the gene/vector/gene product Replication incompetence or competence of a vector Potential for recombination or re-assortment Altered expression of (a) host gene(s) Biodistribution to target/non-target organ(s)/tissue(s)/cell(s) Known interactions with concomitant treatments or known interactions associated with previous exposure to potent agents (chemotherapy, radiotherapy, etc.)

#### Efficacy Issues

One of the biggest issues preventing candidate GTMPs from reaching further development phases is the low efficacy/treatment failure likely related to poor transduction rate (84, 107).

Generally, viral vectors offer higher transduction efficiency and long-term gene expression, when compared with non-viral vectors (15). For instance, AAV2 was the first discovered adeno-associated virus serotype used in early neurodegenerative disorder studies, due to its high neurotropism. Direct injection in the brain parenchyma represents an advantage when compared with systemic administration since it overcomes the need of the vector to pass the blood–brain barrier. In addition, neurodegenerative disorders are often multifocal, affecting several central nervous system (CNS) structures. Widespread CNS distribution of the vector is essential for high treatment efficacy. However, after direct brain administration of the vector it was noted that AAV2 action was limited to the site of injection. Rather than having a strong transduction efficiency throughout the CNS, AAV2 was only able to transduce cells in a limited area. This seemed to be partially related to binding of extracellular matrix components which would prevent intracellular intake (37, 40, 93).

Viral tropism is the affinity to a specific cell or tissue. In recombinant vectors, the tropism is highly dependent on the capsid proteins. Improvement of transgene expression can be accomplished by using a vector with natural tropism for the target cell or engineering the vector’s surface to change the original tropism to the desired target cell (pseudotyping). The latter consists of introducing viral genetic content into a different envelope or altering any capsid protein (55).

A great example of viral pseudotyping is gene therapy development in CF. Direct airway drug delivery encounters a number of challenges such as low availability of relevant vector receptors, short contact time between vector and epithelium, and the barrier function of airway mucus (123). Lentiviral vectors are quite efficient in gene transduction. However, these do not have any natural lung tropism, as opposed to Sendai virus. Pseudotyping with the fusion (F) and HN protein from Sendai virus is a strategy to overcome lentivirus’ natural tropism (72). A recent study showed that the F/HN-pseudotyped lentivirus had significantly greater *in vitro* transduction efficiency when compared with GL67A, the most efficient non-viral vector (124).

Another major hurdle for efficient gene transduction is the endogenous presence of Nab, either against the viral vector or the transgene product. Generally, these antibodies specifically recognize viral capsid proteins, preventing infection. This is of particular importance in therapeutic vectors since these are produced from viruses and preexisting humoral immunity may be an issue not only because it prevents transduction but also because it limits the gene therapy product administration more than once (36, 55). On the other hand, antibodies against the transgene product may result in recruitment of immune cells to the therapeutic product production site with consequent inactivation of the protein (55).

In Glybera, limited efficacy was shown in pivotal studies, especially 1 year after administration, which is not compatible with the intended one-time treatment administration of the GTMP, as a sustained therapeutic effect was not obvious. Viability of retreatment with gene therapy may be achieved by using different serotype vectors, less likely to infect humans. A second administration may be possible if a vector derived from a different serotype is used (58).

Possible strategies to overcome humoral immunity in systemic gene transfer include:

1. Select subjects with low-to-undetectable anti-vector Nab
2. Administer higher vector doses (which may have an impact on safety events)
3. Use empty capsids to adsorb anti-vector antibodies thus allowing transduction
4. Administer immune suppression to prevent or eradicate humoral immune responses
5. Switch vector serotype or engineer vector capsids that are less susceptible to Nab
6. Use repeated plasma exchange cycles to adsorb immunoglobulins and therefore reduce the anti-vector antibody titer (66).

However, in some cases, the low transduction rate is more than enough to have positive clinical results. In hemophilia B, gene therapy administration resulted in less than 10% of normal concentration of the missing clotting factor. This brought significant clinical benefit to a point where a proportion of the treated patients no longer needed artificial clotting factor replacement therapy (125).

#### Drug Development Issues (Non-Clinical and Scale-up)

Because of its unique set of characteristics, the non-clinical development package of a GTMP is rather more complex than conventional medicinal products. Regulators soon recognized that ICH M3 (R2), the general guidance for non-clinical development requirements of new drugs, was inadequate in several aspects when discussing GTMPs. Therefore, the EMA released in 2006 a scientific guideline which details the non-clinical studies required before first clinical use specifically targeted at GTMPs (126). One of the most important differences is that the applicant is expected to have data on the vector particle/delivery system and on the therapeutic transgene(s) as included in the GTMP. The regulators are open to accept data obtained from other similar products. For example, if the same vector is used between two gene therapy candidates with a different transgene product, then the non-clinical studies on the vector can be used, although this may generally not be enough to support first clinical use.

This approach is currently being explored by a number of companies. For instance, Glybera’s UniQure offers a modular AAV-based viral vector platform. Theoretically, the same viral vector could be used to treat different diseases, according to the disease-specific gene content. The greatest advantage would be to have a less extensive preclinical development package reducing time and cost when seeking regulatory approval (127).

Finding adequate animal models may also be an additional challenge and when these are not representative of the clinical situation, regulators encourage the use of homologous animal models (126). Several studies revealed that gene delivery in animal models does not always match clinical setting, from different immune responses to unmatched vector tropism (2).

In trials involving recombinant AAV, an immunological response in humans was observed, which was not seen in the corresponding animal models. This resulted in expression of transgene product levels lower than expected. For example, in a clinical trial for hemophilia patients where FIX was delivered to patients *via* AAV2 vector, two subjects developed an unexpected T cell response to the vectors capsid 4–6 weeks after treatment administration (128). The FIX transgene expression declined to baseline values and around the same time there was an elevation in the hepatic transaminases, suggesting a destruction of transduced hepatocytes. This had not been seen in animal studies. The authors suggested this event was related to cytotoxic T lymphocyte response to the vectors capsid, highlighting that humans are naturally infected by AAV, which is not the case for murine models (128–130).

In spite of the widespread use of rodent models, larger animal models such as non-human primates have proved to be more valuable when it comes to clinical translation, especially regarding toxicology and pharmacokinetics (2).

Manufacturing of gene therapy products is an additional complexity factor. From a regulatory standpoint, these products need to comply with additional guidelines. In Europe, the note for guidance which details the quality aspects of GTMPs (131) was developed in 2001, several years before the implementation of the ATMP law or the CAT, though a revision was made in 2015 (132).

In general, non-viral vectors are more straightforward to produce since they are synthetically developed as opposed to viral vectors (121). In a very simplistic approach, the manufacturing method of a viral vector includes upstream (i.e., the vector assembly) and downstream processes (i.e., vector purification) (133).

The manufacturing process should be GMP compliant, clearly described and performed in certified GMP facilities. For the starting materials, demonstrated evidence on source, quality and control is needed, for both chemical reactants and bacterial/cell/virus seed. On the other hand, the drug substance (i.e., genetic content) should have an extensive genotypic and phenotypic characterization. Its biologic activity should be tested through assessment of the level of transgene expression. Presence of contaminant substances to detect both product-related and process-related impurities (e.g., remaining solvent from purification process) should be carefully determined (132).

Whereas cost and the time are objective parameters in evaluating process efficiency, determining the quality of the production of a recombinant viral vector is not straightforward. Due to the limited experience and low number of approved gene therapy products, vector analytics are not standardized, and contaminants that are present could be completely different among different processes (e.g., residual helper virus versus residual plasmid sequences, human cells versus insect cells versus animal cells). Moreover, assays to test gene therapy products in respect to quality, safety, and efficacy must be developed and validated, which is an additional time consuming task (133, 134). From a quality point of view, *ex vivo* modified cells represent an even higher complexity degree, whether allogenic or autologous cells are used.

Any changes in manufacturing methods may require an assessment of comparability to ensure that these changes have not affected the safety, identity, purity or efficacy of the product (135).

Due to its unique characteristics, gene therapy products require an environmental risk assessment/shedding studies, which intend to collect information about the likelihood of transmission to untreated individuals and measures to prevent such transmission. Shedding is the excretion/secretion of viral particles or bacteria that could be transmitted to other individuals than the patient (135).

Generally, vector manufacturing systems often provide relatively low yields, making clinical administration or non-clinical studies in large animal models quite difficult. Over the past few years, many research groups focused on improving manufacturing processes toward a better up scaling of the product (121). Grieger’s group developed a strategy based on triple transfection for the production of AAV vectors (136). HEK293 packaging cell line unit is used as a basis where three different plasmids are added: a replication (Rep) and Capsid (Cap) plasmid, the desired recombinant vector genome plasmid, and a helper plasmid expressing adenoviral genes. AAV needs a helper virus, such as an AdV or a herpes simplex virus, for adequate replication. By using the third plasmid, addition of the helper virus is unnecessary and the biological hazard of the manufacturing process is reduced.

HEK293 cells are cultured in adherence using bovine serum-based growth media which means that an extensive area would be required to obtain good vector yields. However, Grieger’s group addressed this challenge by developing a method where the cells grow in suspension in serum-free media, within 20 l bioreactors. The safety of the process was increased since the source of adventitious agents was removed, with reduced manufacturing costs. Conversely, larger scale up (to bioreactor with over 200 l) has not yet been demonstrated.

When using HEK293 cells for rAAV production the very low yield is a major limitation. Recombinant baculovirus and insect cells may be an attractive alternative. In 2002, Urabe’s team co-infected insect *Sf9* cells with 3 recombinant baculovirus with positive results. Comparing to vectors produced *via* HEK293 cells, the yield was several times higher and the resulting rAAVs were identical between the two processes (137). In the last few years, some research groups focused their work in fine tuning this process. Mietzsch’s group developed the OneBac in 2014, a system based on insect *Sf9* cell lines containing silent copies of AAV serotypes 1–12 rep and cap genes. Cell induction takes place upon infection with a single baculovirus, carrying the rAAV genome. Besides being a scalable and high-titer production method, the greatest advantage of OneBac is to allow production of a broad spectrum of AAV serotypes (138).

The downstream purification process many include centrifugation and chromatography to remove the empty capsids, which are critical in reducing immune responses due to capsid antigens. As expected, the centrifugation of large volumes is time consuming and a hurdle in up scaling (133, 136).

#### Ethical Conflicts

The discussion on the bioethical hurdles of gene therapy is extensive and focuses on the controversial results that might come from using gene manipulation in both patients and healthy individuals.

Currently, at least in the Western countries, clinical use of gene therapy is limited to somatic cells for the treatment of a specific disease. In a consensus document from the Council of Europe’s Convention on Human Rights and Biomedicine from 1997 it is defined that “*An intervention seeking to modify the human genome may only be undertaken for preventive, diagnostic, or therapeutic purposes and only if its aim is not to introduce any modification in the genome of any descendants*.” Therefore, the use of gene therapy in germline cells with corresponding genetic modification of human gametes or embryos, is not allowed (139).

The discovery of more advanced gene editing tools such as CRISPR/Cas9 technology, transformed the otherwise academic and theoretical debate of germline genetic manipulation into an actual possibility. The CRISPR/Cas9 technology was used in recent experiments where human germline cells were genetically manipulated, by a Chinese research group (140). Almost as a response to this paper, the members of the Organizing Committee for the International Summit on Human Gene Editing published a summit statement where it is highlighted that *in vitro* research including human germline manipulation is acceptable as long as the modified cells are not used to establish a pregnancy (139). To obtain strong and reliable safety and efficacy data, this would require the study of many generations. In 1985, French Anderson defined three conditions that should be met before any attempt to undergo germline gene therapy in humans, which are still valid and up-to-date:

1. Considerable and well-built previous experience with somatic cell gene therapy in humans proving safety and efficacy of the approach
2. Adequate animal research that set up the reproducibility, reliability, and safety of germline therapeutic interventions and
3. The informed public approval of the procedure, since this will impact generations to come and therefore the society as a whole (141).

Another important topic to address is the potential of using gene therapy for purposes other than disease treatment, such as enhancement of genetic engineering or eugenetics. Enhancement of genetic engineering refers to adding a single gene or making changes in a single gene in healthy individuals, while eugenetics can be defined as the attempt to change or improve complex human traits, related to a broader number of genes; for example, personality, intelligence, character. Consequences of such approaches are yet to be determined, in terms of safety or misuse. In this context, the widespread use of gene therapy may have the potential to make society less accepting of people who are different (139, 141, 142).

Patient access to GTMPs raises an additional bioethical issue related to the affordability of these new innovative and potentially curing drugs. Economic difficulties, particularly with regard to unbalanced wealth distribution, may restrict the use of gene therapy products to those who are able to afford them. Glybera, the first gene therapy to be commercially approved in Europe, set its market price at around a million euros (US$1.1 million) per treatment. The projected price for Kymriah is set at 475 thousand US$ (143). For *ex vivo* gene therapies, where patients own cells are modified and then reinfused back into circulation, highly personalized and individualized manufacturing are required, potentially increasing even more the drug cost. Gene therapies have the potential to provide substantial, lifelong benefit to the patient on a single administration, which may compensate the cost of the standard treatment of the condition and its complications (41).

## Conclusion

Even though the first trials with GTMPs occurred in the 1970s, in Europe, the ATMP regulation was only fully implemented in 2009, highlighting that science moves faster than regulators. Since the CAT was fully in place, to date, only eight ATMPs were granted MA. From those, three are GTMPs. The EMA acknowledges the great potential of these therapies in addressing high unmet medical needs and a strong effort is underway in promoting its development.

A comprehensive understanding of GTMP drug development challenges is critical when designing development programs and obtaining marketing authorization. Careful choice of vector is fundamental in effective gene delivery in addition to overcoming immunogenic and oncogenic safety issues and the recurrently observed poor efficacy. Monogenic diseases represent the most successful clinical application, although the use of gene therapy in more complex diseases is also being tested, as well as alternative strategies such as RNA targeting or targeted gene editing.

These therapies are likely to have a strong impact over the public health landscape. Ethical implications related to the use of gene therapy need to be fully understood. In addition, the anticipated high price of ATMPs is expected to generate added controversy. Establishment of a viable business model is essential as the field may not survive without it. Given the high number of research projects in the field and the incredible promising profile of such therapies it is expected that more and more discussion around GTMPs will take place.

## Author Contributions

MC conducted the bibliographic search, analyzed the data, and drafted the manuscript. BS and AM analyzed and reviewed the data and edited the manuscript.

## Conflict of Interest Statement

The authors declare that the research was conducted in the absence of any commercial or financial relationships that could be construed as a potential conflict of interest.
